# *Populus* resequencing: towards genome-wide association studies

**DOI:** 10.1186/1753-6561-5-S7-I21

**Published:** 2011-09-13

**Authors:** Gerald Tuskan, Gancho Slavov, Steve DiFazio, Wellington Muchero, Ranjan Pryia, Wendy Schackwitz, Joel Martin, Daniel Rokhsar, Robert Sykes, Mark Davis, Michael Studer, Charles Wyman

**Affiliations:** 1 Oak Ridge National Laboratory, USA; 2 West Virginia University, USA; 3 Joint Genome Institute, USA; 4 National Renewable Energy Laboratory, USA; 5 Univeresity of California Riverside, USA

## 

Genome-wide association studies (GWAS) have been used to identify regions of the genome related to various phenotypes in humans, corn, rice and cattle. Successful application of this approach to bioenergy crops such as *Populus* requires 1) an appropriate mapping population, 2) high-quality phenotypic data and 3) informative genotypic data. With the goal of reducing the recalcitrance of lignocellulosic biomass for economic production of biofuels and understanding basic mechanisms of cell wall formation in *Populus* we established 4 clonally replicated common gardens experiments each with 1100 native *P*. *trichocarpa* genotypes collected from along the northwest coast of the U.S. and Canada. A high-throughput phenotyping pipeline was developed to measure cell wall chemistry, pretreatment response and enzymatic sugar release. Initially 18 genotypes were resequenced to an average 30X depth in order to design a SNP array to test for statistical association using MMAX and PCA methods of testing among ca. 2500 candidate genes. Genetic structure and linkage disequilibrium (LD) was assessed using SSR and SNP markers. Outlying genotypes were excluded from the analyses and estimates of LD were used to design the bead array. Candidate genes were selected based on QTL intervals, expression profiling within developing xylem and expert opinion. MMAX and PCA results revealed similar significant associations for all measured phenotypes and several SNPs within the candidate gene set explain a relatively high degree of the phenotypic variance. As a result, resequencing has continued in order to conduct GWAS in *Populus*; the complete set of 1100 genotypes will be complete in 2012.

